# Two Novel Two-Stage Direction of Arrival Estimation Algorithms for Two-Dimensional Mixed Noncircular and Circular Sources

**DOI:** 10.3390/s17061433

**Published:** 2017-06-18

**Authors:** Heping Shi, Wen Leng, Zhiwei Guan, Tongzhi Jin

**Affiliations:** 1 School of Automotion and Transportation, Tianjin University of Technology and Education, Tianjin 300222, China; shiheping@tju.edu.cn; 2 School of Electrical and Information Engineering, Tianjin University, Tianjin 300072, China; lengwen@tju.edu.cn (W.L.); jin1218@tju.edu.cn (T.J.)

**Keywords:** 2D direction-of-arrival estimation, noncircular signal, circularity difference, small angle separation

## Abstract

This paper addresses the two-dimensional (2D) direction-of-arrival (DOA) estimation problem with two novel methods for mixed noncircular and circular signals. The first proposed method is named the two-stage direction-of-arrival matrix (TSDOAM) method, and the other is called the two-stage rank reduction (TSRARE) method. The proposed methods utilize both the circularity and the direction-of-arrival differences between the noncircular and circular sources to estimate the 2D directions-of-arrival (DOAs). The maximum detectable 2D angle parameters of the TSDOAM and TSRARE methods are twice those of the existing methods. Moreover, the TSRARE method can detect more incident signals than the TSDOAM method due to the array aperture of two parallel uniform linear arrays (ULAs) being fully utilized. Simulation results show that compared to the existing methods for the small angle separation of 2D directions-of-arrival, the two proposed methods perform well in terms of the signal-to-noise ratio (SNR) and snapshots.

## 1. Introduction

Recently, the noncircularity of incident signals has been widely reported in the field of array signal processing, including direction-of-arrival (DOA) estimation [1,2,3,4,5,6,7,8,9,10] and beamforming [11,12,13] to improve the performance of direction-of-arrival estimation accuracy and beamformers. The aforementioned direction-of-arrival estimation algorithms are mainly focused on the one-dimensional (1D) domain. However, in practice, two-dimensional (2D) direction-of-arrival estimation with various array structures, such as two-parallel arrays [14,15,16,17,18,19], L-shaped arrays [20,21,22,23,24,25,26,27], and a uniform rectangular array [28,29,30], are closer to the actual situation.

In order to improve the direction-of-arrival estimation performance, many effective noncircular algorithms for 2D directions-of-arrival have been presented in [31,32,33]. In [31], Liu et al. proposed an extended rank reduction (ERARE) method with noncircular information exploited for two-parallel uniform linear arrays (ULAs) which achieved an improved estimation accuracy compared to [15]. Based on [25], the conjugate information of the observed data was utilized to realize a better 2D direction-of-arrival estimation [32]. use of the conjugate information of the observed data to realize a better 2D direction-of-arrival estimation. A method that applied noncircular direction finding to the hexagonally-shaped electronically steerable parasitic antenna radiator (ESPAR) array was presented in [33], and the Cramér–Rao bound (CRB) was analyzed for comparison. However, the aforementioned algorithms cannot cope with the direction-of-arrival estimation problem for the mixed noncircular (e.g., binary phase shift keying, BPSK) and circular (e.g., quaternary phase shift keying, QPSK) signal scenario. Although Yin’s method [15] and Xia’s method [16] could be applicable to the above-mentioned mixed signal scenario, the distinguishable signals were less than the array elements.

In [34,35,36], direction-of-arrival estimation schemes for joint noncircular and circular signal estimation were proposed for 1D direction finding. In [34], Gao. et al. proposed a method that constructed a new data vector with the original data and the conjugate ones to form two estimators for noncircular and circular signal finding, respectively. However, the method in [34] cannot deal with the coincident directions-of-arrival of noncircular and circular signals, and its estimation performance degraded severely in small angle separation . In addition, the maximum number of detected signals was still limited. An improved method was presented in [35] to solve the above problems, which adopted the direction-of-arrival circularity difference rather than the direction-of-arrival difference between the noncircular and circular signals to estimate the direction-of-arrival. Nevertheless, the direction-of-arrival estimation performance dropped due to few observed data being available. In [36], a sparse representation method for mixed signals was proposed by exploiting overcomplete dictionaries that were subject to the sparsity constraint to jointly represent the covariance and elliptic covariance matrices of the array output. However, for 2D situations, there are few research works for joint noncircular and circular signal direction finding. Additionally, much of the work in array processing has also been focused on optimization problems, such as genetic algorithms [37,38,39,40].

In this paper, inspired by the method in [35], two novel 2D direction-of-arrival estimation algorithms using two parallel ULAs with a two-stage direction-of-arrival matrix (TSDOAM) method and a two-stage rank reduction (TSRARE) method, separately, are proposed for mixed noncircular and circular signals estimation. The direction-of-arrival circularity difference rather than the direction-of-arrival difference between the noncircular and circular signals for the 2D directions-of-arrival’s estimation is utilized in the two proposed methods. The maximum number of distinguished mixed signals of the two proposed methods are identified compared to the conventional methods, which show that the detected number of the signals is more than that of the array elements. Moreover, when both of the 2D directions-of-arrival are incoming from small angle separation—even when both of them are from the overlapping direction—the estimation accuracy of the two proposed methods is better than Yin’s and Xia’s method.

The rest of this paper is organized as follows. The array signal model is introduced in Section 2. The TSDOAM and TSRARE methods are described in detail in Section 3. The maximum number of detective mixed signals is analyzed in Section 4. Simulation results are presented to verify the performance of the two proposed methods in Section 5. Conclusions are drawn in Section 6.

Throughout this paper, the following notations are used. ${\left(\cdot \right)}^{*}$, ${\left(\cdot \right)}^{T}$, ${\left(\cdot \right)}^{-1}$, ${\left(\cdot \right)}^{+}$, and ${\left(\cdot \right)}^{H}$ represent conjugation, transpose, inverse, pseudo-inverse, and conjugate transpose, respectively. E(·) indicates the expectation operator; arg(·) is to get the phase; diag(·) stands for the diagonalization operation of a vector.

## 2. Array Signal Model

**Figure 1:**
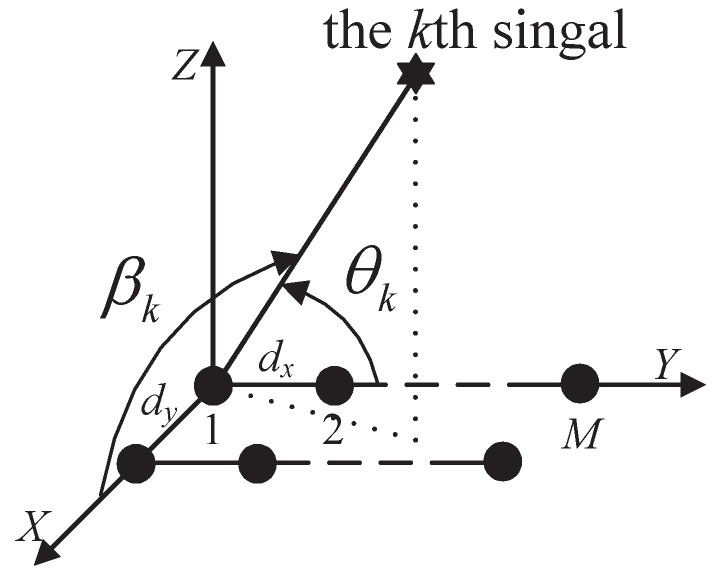
The geometry configuration of the array.

As shown in Figure 1, suppose that there are $K=K_{n}+K_{c}$ (assume the number of mixed signals is known to the receiver) uncorrelated far-field sources that are mixed $K_{n}$ noncircular sources $s_{n,k}\left(t\right)$ and $K_{c}$ circular sources $s_{c,k}\left(t\right)$ from direction $(\theta_{k},\beta_{k}),k=1,2,\ldots ,K$, impinging on two parallel ULAs with each array having *M* elements. The distance between the two arrays is λ/2, denoted as $d_{y}$, and the interelement spacing $d_{x}$ on each array is also λ/2, where λ is the wavelength of the incident waves. The additive noises of two ULAs are circular Gaussian noises, which are uncorrelated with the incoming signals.

The observed data vectors $X\left(t\right)={\left[x_{1}\left(t\right),x_{2}\left(t\right),\ldots ,x_{M}\left(t\right)\right]}^{T}$ and $Y\left(t\right)={\left[y_{1}\left(t\right),y_{2}\left(t\right),\ldots ,y_{M}\left(t\right)\right]}^{T}$ from two parallel ULAs are given by:

(1) $$X\left(t\right)=AS\left(t\right)+N_{x}\left(t\right)=A_{n}S_{n}\left(t\right)+A_{c}S_{c}\left(t\right)+N_{x}\left(t\right)$$

(2) $$Y\left(t\right)=ABS\left(t\right)+N_{y}\left(t\right)=A_{n}BS_{n}\left(t\right)+A_{c}BS_{c}\left(t\right)+N_{y}\left(t\right)$$

where A is called the steering vector with each column denoted $a(\theta_{k})$ and $a\left(\theta_{k}\right)={\left[a_{0}\left(\theta_{k}\right),\ldots ,a_{M-1}\left(\theta_{k}\right)\right]}^{T}$, whose element can be expressed as $a_{i}\left(\theta_{k}\right)=e^{-j\frac{2\pi }{\lambda }d_{x}\left(i\right)\cos\theta_{k}}$. B(β) is termed as the steering element matrix with the expression $B=\mathrm{diag}[v\left(\beta_{1}\right),v\left(\beta_{2}\right),\ldots ,v\left(\beta_{K}\right)]$, and the element $v(\beta_{k})$ has the form of $e^{j\frac{2\pi }{\lambda }d_{y}\cos\beta_{k}}$. $S\left(t\right)=[s_{1}\left(t\right),s_{2}\left(t\right),\ldots ,s_{K}\left(t\right)]$ denotes the radiating signal vector. $N_{x}\left(t\right)={\left[n_{x,1}\left(t\right),\ldots ,n_{x,M}\left(t\right)\right]}^{T}$ and $N_{y}\left(t\right)={\left[n_{y,1}\left(t\right),\ldots ,n_{y,M}\left(t\right)\right]}^{T}$ represent the circular Gaussian noise vectors of the two arrays, respectively.

## 3. The Two Proposed Algorithms

### 3.1. The TSDOAM Method

A novel method called the TSDOAM method with the two-stage direction-of-arrival matrix (DOAM) method and direction-of-arrival circularity difference, is proposed in this part. According to Equations (1) and (2), and based on the assumption that the noise and the signals are uncorrelated and that the mixed signals are also uncorrelated, the auto-covariance matrix $R_{xx}$ and cross-covariance matrix $R_{yx}$ can be written, respectively, as follows.

(3) $$R_{xx}=E\left[X\left(t\right)X^{H}\left(t\right)\right]=A_{n}R_{n}A_{n}^{H}+A_{c}R_{c}A_{c}^{H}+\sigma^{2}I_{xx}$$

(4) $$R_{yx}=E\left[X\left(t\right)Y^{H}\left(t\right)\right]=A_{n}\Phi_{n}R_{n}A_{n}^{H}+A_{c}\Phi_{c}R_{c}A_{c}^{H}$$

where $A_{n}$ and $A_{c}$ denote the steering matrices associated with noncircular and circular signals, separately. $\sigma^{2}$ is the variance of the circular Gaussian noises, and $R_{n}$ and $R_{c}$ have the form of $R_{n}=\mathrm{diag}\{E\left[s_{n,1}s_{n,1}^{*}\right],...,E\left[s_{n,K_{n}}s_{n,K_{n}}^{*}]\right\}$ and $R_{c}=\mathrm{diag}\{E\left[s_{c,1}s_{c,1}^{*}\right],...,E\left[s_{c,K_{c}}s_{c,K_{c}}^{*}\right]\}$, respectively.

In practice, non-circularity and circularity are important properties of a random variable; their concept comes directly from the geometrical interpretation of a complex random variable. The source would be called a circular source if its statistical characteristics have a rotational invariance characteristic; otherwise, it would be called a noncircular source. Based on this, we only consider the rotational invariance characteristic of the first- and second-order statistical properties of the sources. For a complex random source $s_{k}$, we define $E[s_{k}]$, $E[s_{k}s_{k}^{*}]$, and $E[s_{k}^{2}]$ as the mean, the covariance, and the elliptic covariance of the source $s_{k}$, respectively. For an arbitrary phase $\varphi_{k}$ as follows:

(5) $$E\left[s_{k}e^{j\varphi_{k}}\right]=E\left[s_{k}\right]$$

(6) $$E\left[s_{k}e^{j\varphi_{k}}{\left(s_{k}e^{j\varphi_{k}}\right)}^{*}\right]=E\left[s_{k}s_{k}^{*}\right]$$

(7) $$E\left[s_{k}e_{k}^{j\varphi }\left(s_{k}e_{k}^{j\varphi }\right)\right]=E\left[s_{k}^{2}\right]$$

If the source’s first- and second-order statistical properties are rotationally invariant, the source is identified as circular; otherwise, it is determined to be noncircular. Therefore, the elliptic auto-covariance matrix $R_{xx}^{\prime }$ can be expressed as follows:

(8) $$R_{xx}^{\prime }=E\left[X\left(t\right)X^{T}\left(t\right)\right]=A_{n}R_{n}^{\prime }A_{n}^{T}+A_{c}R_{c}^{\prime }A_{c}^{T}+E\left[N_{x}\left(t\right)N_{x}^{T}\left(t\right)\right]$$

Notice that for a complex circular random variable *h*, E[hh]=0 [6]. Therefore, the circular component and the circular Gaussian noise component of Equation (8) both equal zero, and the elliptic auto-covariance matrix $R_{xx}^{\prime }$ can be rewritten as:

(9) $$R_{xx}^{\prime }=A_{n}R_{n}^{\prime }A_{n}^{T}$$

where $R_{n}^{\prime }=\mathrm{diag}\{E\left[s_{n,1}s_{n,1}\right],...,E\left[s_{n,K_{n}}s_{n,K_{n}}\right]\}$.

Similarly, the elliptic cross-covariance matrix $R_{yx}^{\prime }$ is computed as follows:

(10) $$R_{yx}^{\prime }=A_{n}\Phi_{n}R_{n}^{\prime }A_{n}^{T}$$

We then estimate the 2D directions-of-arrival of noncircular signals with Equations (9) and (10). First, let $\left\{\eta_{n,1},...,\eta_{n,K_{n}}\right\}$ and $\left\{v_{n,1},...,v_{n,K_{n}}\right\}$ be the eigenvalues and corresponding eigenvectors of $R_{xx}^{\prime }$, respectively, namely:

(11) $$R_{xx}^{\prime }=\sum_{k=1}^{K_{n}}\eta_{n,k}v_{n,k}v_{n,k}^{H}$$

The pseudo-inverse of $R_{xx}^{\prime +}$ is:

(12) $$R_{xx}^{\prime +}=\sum_{k=1}^{K_{n}}\eta_{n,k}^{-1}v_{n,k}v_{n,k}^{H}$$

Due to $R_{n}^{\prime }$ being a diagonal matrix and $A_{n}$ a column full-rank matrix, we attain the following formula with Equation (9):

(13) $$R_{n}^{\prime }A_{n}^{T}={\left(A_{n}^{H}A_{n}\right)}^{-1}A_{n}^{H}R_{xx}^{\prime }$$

Combining Equation (13) with (10), we obtain an alternative expression of $R_{yx}^{\prime }$:

(14) $$\begin{array}{cc} R_{yx}^{\prime } & =A_{n}\Phi_{n}R_{n}^{\prime }A_{n}^{T}\;\\ & =A_{n}\Phi_{n}{\left(A_{n}^{H}A_{n}\right)}^{-1}A_{n}^{H}R_{xx}^{\prime } \end{array}$$

Right-multiplying both sides of Equation (14) by $R_{xx}^{\prime +}A_{n}$, we get:

(15) $$\begin{array}{cc} R_{yx}^{\prime }R_{xx}^{\prime +}A_{n} & =A_{n}\Phi_{n}R_{n}^{\prime }A_{n}^{T}R_{xx}^{\prime +}A_{n}\\ & =A_{n}\Phi_{n}{\left(A_{n}^{H}A_{n}\right)}^{-1}A_{n}^{H}R_{xx}^{\prime }R_{xx}^{\prime +}A_{n} \end{array}$$

Substituting Equations (11) and (12) into Equation (15),

(16) $$\begin{array}{cc} R_{yx}^{\prime }R_{xx}^{\prime +}A_{n} & =A_{n}\Phi_{n}{\left(A_{n}^{H}A_{n}\right)}^{-1}A_{n}^{H}\left(\sum_{k=1}^{K_{n}}\eta_{n,k}v_{n,k}v_{n,k}^{H}\right)\left(\sum_{k=1}^{K_{n}}\eta_{n,k}^{-1}v_{n,k}v_{n,k}^{H}\right)A_{n}\\ & =A_{n}\Phi_{n}{\left(A_{n}^{H}A_{n}\right)}^{-1}A_{n}^{H}\left(\sum_{k=1}^{K_{n}}v_{n,k}v_{n,k}^{H}\right)A_{n} \end{array}$$

From Equations (1) and (2), we can see that the dimensions of the observed data vectors X(t) and Y(t) are both M×1. Then, based on Equations (3) and (4), it is easy to know that the dimensions of the auto-covariance matrix $R_{xx}$ and cross-covariance matrix $R_{yx}$ are both M×M. From Equation (8), we can see that the dimension of the elliptic auto-covariance matrix $R_{xx}^{\prime }$ is also M×M. Therefore, $R_{xx}^{\prime }$ is a square matrix. Specifically for the first proposed method in this paper, the $R_{xx}^{\prime +}$—which is the pseudo-inverse of $R_{xx}^{\prime }$—is equivalent to the inverse of $R_{xx}^{\prime }$. That is to say, the $R_{xx}^{\prime }$ and $R_{xx}^{\prime +}$ in the first proposed method are both square matrices. Moreover, the diagonal elements of $R_{xx}^{\prime +}$ are nonzero elements , and $R_{xx}^{\prime +}$ is full rank. Therefore, $\sum_{k=1}^{K_{n}}v_{n,k}v_{n,k}^{H}$ is an identity matrix, and Equation (16) can be simplified as:

(17) $$\begin{array}{cc} R_{yx}^{\prime }R_{xx}^{\prime +}A_{n} & =A_{n}\Phi_{n}{\left(A_{n}^{H}A_{n}\right)}^{-1}A_{n}^{H}A_{n}\\ & =A_{n}\Phi_{n} \end{array}$$

From Equation (17), 2D directions-of-arrival of noncircular signals—which are obtained by performing eigenvalue decomposition (EVD) of $R_{yx}^{\prime }R_{xx}^{\prime +}$, denoted as the direction-of-arrival matrix, lie in $A_{n}$ and $\Phi_{n}$, respectively.

(18) $$R_{yx}^{\prime }R_{xx}^{\prime +}=\sum_{k=1}^{K_{n}}\xi_{n,k}u_{n,k}u_{n,k}^{H}$$

where $\xi_{n,k}$ and $u_{n,k}$ are the eigenvalues and the corresponding eigenvectors of $R_{yx}^{\prime }R_{xx}^{\prime +}$, respectively. It can be verified that the spanned subspace from the steering matrix $A_{n}$ and the signal subspace $U_{n}=\left[u_{n,1},\ldots ,u_{n,K_{n}}\right]$ are the same.

Unlike Yin’s method obtaining the 1D angle by the spectrum search with a certain region (which entailed high complexity), here, define $h_{n,k}=u_{n,k}/u_{n,k}\left(1\right)$; we can get:

(19) $$\kappa_{n,k}=\frac{1}{M-1}\sum_{i=1}^{M-1}arg\left[\frac{h_{n,k}\left(i+1\right)}{h_{n,k}\left(i\right)}\right].$$

Integrating the expression $a_{i}\left(\theta_{n,k}\right)$ and $v(\beta_{n,k})$ with Equations (18) and (19), the estimated 2D directions-of-arrival of noncircular signals are achieved as follows:

(20) $$\theta_{n,k}=\arccos(-\frac{\lambda }{2\pi d_{x}}\kappa_{n,k}),$$

(21) $$\beta_{n,k}=\arccos\left[\frac{\lambda }{2\pi d_{y}}arg\left(\xi_{n,k}\right)\right].$$

In the next stage, the 2D direction-of-arrival of the circular signals can be obtained with the estimates $\theta_{n,k}$ and $\beta_{n,k}$ above.

With Equation (9), $R_{n}^{\prime }$ can be estimated as:

(22) $$R_{n}^{\prime }=A_{n}^{+}R_{xx}^{\prime }{\left(A_{n}^{T}\right)}^{+}$$

where $A_{n}^{+}={\left(A_{n}^{H}A_{n}\right)}^{-1}A_{n}^{H}$ and ${\left(A_{n}^{T}\right)}^{+}=A_{n}^{*}{\left(A_{n}^{T}A_{n}^{*}\right)}^{-1}$ [41].

Let the *k*th diagonal element of $R_{n}^{\prime }$ be $R_{n}^{\prime }\left(k,k\right)$; we get:

(23) $$R_{n}^{\prime }\left(k,k\right)=E\left[s_{n,k}s_{n,k}\right].$$

here, we assume that the noncircular signals are BPSK-modulated signals. Therefore, $E\left[s_{n,k}s_{n,k}\right]=\sigma_{n,k}^{2}e^{j\varphi_{n,k}}$, where $\sigma_{n,k}^{2}=E\left[s_{n,k}s_{n,k}^{*}\right]$ and $\varphi_{n,k}$ are the noncircular phases. It is easily deduced that $\sigma_{n,k}^{2}=\left|R_{n}^{\prime }\left(k,k\right)\right|$. Due to $R_{n}$ being diagonal, it follows that:

(24) $$\begin{array}{cc} R_{n} & =\mathrm{diag}[\sigma_{n,1}^{2},\ldots ,\sigma_{n,K_{n}}^{2}]\\ & =\mathrm{diag}[|R_{n}^{\prime }\left(1,1\right)|,\ldots ,|R_{n}^{\prime }\left(K_{n},K_{n}\right)|]. \end{array}$$

Then, let $R_{xx,1}=A_{n}R_{n}A_{n}^{H}$ and $R_{yx,1}=A_{n}\Phi_{n}R_{n}A_{n}^{H}$; we attain:

(25) $$R_{xx}-R_{xx,1}=A_{c}R_{c}A_{c}^{H}+\sigma^{2}I_{xx}.$$

(26) $$R_{yx}-R_{yx,1}=A_{c}\Phi_{n}R_{c}A_{c}^{H}.$$

Define:

(27) $$R_{xx,2}=R_{xx}-R_{xx,1}-\sigma^{2}I_{xx}=A_{c}R_{c}A_{c}^{H}$$

(28) $$R_{yx,2}=R_{yx}-R_{yx,1}=A_{c}\Phi_{c}R_{c}A_{c}^{H}$$

We have another direction-of-arrival matrix $R_{yx,2}R_{xx,2}^{+}$ that is related to circular signals.

Just as the way of attaining the 2D directions-of-arrival of noncircular signals, the 2D directions-of-arrival $\theta_{c,k}$ and $\beta_{c,k}$ of circular signals are obtained with the direction-of-arrival matrix $R_{yx,2}R_{xx,2}^{+}$ using the same direction-of-arrival matrix (DOAM) method.

Until now, the TSDOAM method is summarized as follows.

**Step** **1:** Calculate $R_{xx}$, $R_{yx}$, $R_{xx}^{\prime }$, and $R_{yx}^{\prime }$ from Equations (3)–(10);
**Step** **2:** Execute the EVD of $R_{xx}^{\prime }$ to get its pseudo-inverse matrix $R_{xx}^{\prime +}$;
**Step** **3:** Perform the EVD of $R_{yx}^{\prime }R_{xx}^{\prime +}$ with Equation (18);
**Step** **4:** Attain $\theta_{n,k}$ and $\beta_{n,k}$ using Equations (20) and (21);
**Step** **5:** Construct the matrix $R_{n}^{\prime }$ with the estimate $R_{xx}^{\prime }$ and $A_{n}$;
**Step** **6:** Construct the direction-of-arrival matrix $R_{yx,2}R_{xx,2}^{+}$ with Equations (27) and (28);
**Step** **7:** Repeat **Step 2** to **Step 4** for the $\theta_{c,k}$ and $\beta_{c,k}$.

### 3.2. The TSRARE Method

In this section, in order to make full use of array elements of two ULAs, another novel method called the TSRARE method—which is based on the direction-of-arrival circularity difference and the rank reduction (RARE) method—is proposed in the two-stage estimation procedure.

By concatenating the observed data vectors X(t) and Y(t), we get:

(29) $$\begin{array}{cc} Z(t) & =\left[\begin{array}{c} X(t)\\ Y(t) \end{array}\right]=\left[\begin{array}{c} A\\ AB \end{array}\right]S\left(t\right)+\left[\begin{array}{c} N_{x}\left(t\right)\\ N_{y}\left(t\right) \end{array}\right]\\ & =CS(t)\;+\;N(t). \end{array}$$

where $C=[c\left(\theta_{1},\beta_{1}\right),\ldots ,c\left(\theta_{K},\beta_{K}\right)]$ is termed as the extended steering matrix, and:

(30) $$\begin{array}{cc} c(\theta_{k},\beta_{k}) & =\left[\begin{array}{c} a(\theta_{k})\\ a\left(\theta_{k}\right)v\left(\beta_{k}\right) \end{array}\right]\\ & =\left[\begin{array}{cc} a(\theta_{k}) & 0\\ 0 & a(\theta_{k}) \end{array}\right]\left[\begin{array}{c} 1\\ v(\beta_{k}) \end{array}\right]. \end{array}$$

As the radiating mixed signals are uncorrelated with each other, the conjugated covariance matrix R of Z(t) can be written as:

(31) $$R=E\left[Z\left(t\right)Z^{H}\left(t\right)\right]=C_{n}R_{n}C_{n}^{H}+C_{c}R_{c}C_{c}^{H}+\sigma^{2}I,$$

and the elliptic covariance matrix $R^{\prime }$ of Z(t) is as follows:

(32) $$R^{\prime }=E\left[Z\left(t\right)Z^{T}\left(t\right)\right]=C_{n}R_{n}^{\prime }C_{n}^{T}$$

where $C_{n}$ and $C_{c}$ denote the noncircular and circular extended steering matrix, respectively.

Next, we perform singular-value decomposition (SVD) of $R^{\prime }$ to estimate the 2D directions-of-arrival of noncircular signals as follows:

(33) $$R^{\prime }=\left[U_{n,1}\;\;U_{n,2}\right]\left[\begin{array}{cc} \Lambda_{n} & 0\\ 0 & 0 \end{array}\right]V_{n}^{H}$$

where $\Lambda_{n}$ denotes the $K_{n}\times K_{n}$ diagonal matrix containing $K_{n}$ nonzero singular values in the diagonal. It is verified that all of the columns of $C_{n}$ are orthogonal to all of the columns of $U_{n,2}$; that is,

(34) $$C_{n}^{H}\left(\theta_{n,k},\beta_{n,k}\right)\;U_{n,2}U_{n,2}^{H}C_{n}\left(\theta_{n,k},\beta_{n,k}\right)\;=0,k=1,\ldots ,K_{n}.$$

Associated with Equation (30), Equation (34) can be rewritten as:

(35) $$r^{H}Tr=0$$

where $r={\left[1\;\;v^{T}\left(\beta_{n,k}\right)\right]}^{T}$, and:

(36) $$T\left(\theta_{n,k}\right)=\left[\begin{array}{cc} a^{H}\left(\theta_{n,k}\right)U_{n,1}^{\prime }a\left(\theta_{n,k}\right) & a^{H}\left(\theta_{n,k}\right)U_{n,2}^{\prime }a\left(\theta_{n,k}\right)\\ a^{H}\left(\theta_{n,k}\right)U_{n,3}^{\prime }a\left(\theta_{n,k}\right) & a^{H}\left(\theta_{n,k}\right)U_{n,4}^{\prime }a\left(\theta_{n,k}\right) \end{array}\right]$$

where $U_{n,1}^{\prime }=U_{n,21}U_{n,21}^{H}$, $U_{n,2}^{\prime }=U_{n,21}U_{n,22}^{H}$, $U_{n,3}^{\prime }=U_{n,22}U_{n,21}^{H}$, and $U_{n,4}^{\prime }=U_{n,22}U_{n,22}^{H}$, $U_{n,21}$ and $U_{n,22}$ are obtained by dividing $U_{n,2}$ into the same two-dimensional matrices—namely $U_{n,2}={\left[U_{n,21}^{T}\;\;\;\;U_{n,22}^{T}\right]}^{T}$.

In order to use the RARE method, we define $a(\theta_{n,k})$ as:

(37) $$a\left(b_{n,k}\right)={\left[1,b_{n,k},b_{n,k}^{2},\ldots ,b_{n,k}^{M-1}\right]}^{T}$$

where $b_{n,k}=e^{-j\frac{2\pi }{\lambda }d_{x}\cos\theta_{n,k}}$, and the matrix T is a function of $b_{n,k}$. Then, the 1D directions-of-arrival $\theta_{n,k}$ can be obtained by finding the values of $b_{n,k}$ such that $\det[T\left(b_{n,k}\right)]=0$. Additionally, the polynomial of $b_{n,k}$ has the following form:

(38) $$\det\left[T\left(b_{n,k}\right)\right]=m_{n,1}m_{n,4}-m_{n,2}m_{n,3}$$

where $m_{n,p}=a^{T}\left(1/b_{n,k}\right)U_{n,p}^{\prime }a\left(b_{n,k}\right),\;\;\;\;\;p=1,2,3,4$. $m_{n,p}$ is the polynomial of $b_{n,k}$ whose *l*th coefficient is given by the sum of the elements of the *l*th diagonal of $U_{n,p}^{\prime }$, where l=-M+1,…,M-1. Collecting the coefficients of the polynomial $m_{n,p}$ as a column vector denoted as $\mu_{n,p}={\left[\mu_{np,1},\ldots ,\mu_{np,l},\ldots ,\mu_{np,2M-1}\right]}^{T}$, we get $m_{n,p}=o_{n}\mu_{n,p}$, where $o_{n}=\left[b_{n,k}^{-M+1},\ldots ,1,\ldots ,b_{n,k}^{M-1}\right]$, $\mu_{np,l}\;=\;\sum_{i\;=\;\max[1,M-l\;+\;1]}^{min[M,2M-l]}{[U}_{n,p}^{\prime }{]}_{i,l+i-M}$.

Thus, we have that $m_{n,1}m_{n,4}=o_{n}\mu_{n,1}\mu_{n,4}^{T}o_{n}^{T}$ and $m_{n,2}m_{n,3}=o_{n}\mu_{n,2}\mu_{n,3}^{T}o_{n}^{T}$, and the coefficients of the polynomials $m_{n,1}m_{n,4}$ and $m_{n,2}m_{n,3}$ equal the sum of the antidiagonal elements of the matrix $\mu_{n,1}\mu_{n,4}^{T}$ and $\mu_{n,2}\mu_{n,3}^{T}$, respectively. Let $\delta_{n,f}={\left[\delta_{nf,1},\ldots ,\delta_{nf,l},\ldots ,\delta_{nf,4M-3}\right]}^{T},f=1,2$ be the column vectors of the 4m-3 coefficients of the polynomials $m_{n,1}m_{n,4}$ and $m_{n,2}m_{n,3}$, where $\delta_{nf,l}\;=\;\sum_{i\;=\;\max[1,l-2M\;+\;2]}^{min[2M-1,l]}[\Phi_{n,f}{]}_{i,l-i+1}$, $\Phi_{n,1}\;=\;\mu_{n,1}\mu_{n,4}^{T}$ and $\Phi_{n,2}\;=\;\mu_{n,2}\mu_{n,3}^{T}$.

Hence, Equation (38) can be expressed as:

(39) $$\det\left[T\left(b_{n,k}\right)\right]=\sum_{l=1}^{4M-3}\left(\delta_{n1,l}-\delta_{n2,l}\right)b_{n,k}^{l-(2M-1)}=0.$$

The roots of the polynomial $\det[T\left(b_{n,k}\right)]$ can be computed by exploiting the computationally-efficient polynomial root multiple signal classification (MUSIC) algorithm, and the 1D noncircular angles $\theta_{n,k}$ are obtained as:

(40) $$\theta_{n,k}=\arccos\left[\frac{\lambda }{2\pi d_{x}}arg\left(b_{n,k}\right)\right].$$

Substituting the estimated $\theta_{n,k}$ into Equation (35), we then seek out the minima of the following function [42]:

(41) $$\beta_{n,k}=\min_{\beta_{n}}r^{H}Tr$$

From Equation (41), we obtain $\beta_{n,k}$ that are given by the eigenvector corresponding to the smallest eigenvalue associated with $T(\theta_{n,k})$ as:

(42) $$\beta_{n,k}=\arccos\left[\frac{\lambda }{2\pi d_{y}}arg\left[-a^{H}\left(\theta_{n,k}\right)U_{n,3}^{\prime }a\left(\theta_{n,k}\right)\right]\right].$$

Next, we estimate the 2D directions-of-arrival of circular signals based on the estimates $\theta_{n,k}$ and $\beta_{n,k}$. With Equations (30) and (32), $R_{n,1}^{\prime }$—denoted as the estimated $R_{n}^{\prime }$ in the TSRARE method—can be expressed as:

(43) $$R_{n,1}^{\prime }=C_{n}^{+}R^{\prime }{\left(C_{n}^{T}\right)}^{+}$$

where $C_{n}^{+}={\left(C_{n}^{H}C_{n}\right)}^{-1}C_{n}^{H}$ and ${\left(C_{n}^{T}\right)}^{+}=C_{n}^{*}{\left(C_{n}^{T}C_{n}^{*}\right)}^{-1}$ [41].

Denoting the *k*th diagonal element of $R_{n,1}^{\prime }$ as $R_{n,1}^{\prime }\left(k,k\right)$, we have:

(44) $$R_{n,1}^{\prime }\left(k,k\right)=E\left[s_{n,k}s_{n,k}\right].$$

Similarly, the noncircular signals here are BPSK-modulated signals. It is easily deduced that $\sigma_{n,k}^{2}=\left|R_{n,1}^{\prime }\left(k,k\right)\right|$. Because $R_{n}$ is diagonal, it follows that $R_{n,1}$, which is the estimate of $R_{n}$ as follows:

(45) $$\begin{array}{cc} R_{n,1} & =\mathrm{diag}[\sigma_{n,1}^{2},\ldots ,\sigma_{n,K_{n}}^{2}]\\ & =\mathrm{diag}[|R_{n,1}^{\prime }\left(1,1\right)|,\ldots ,|R_{n,1}^{\prime }\left(K_{n},K_{n}\right)|]. \end{array}$$

Then, let $R_{1}=C_{n}R_{n,1}C_{n}^{H}$; we attain:

(46) $$R-R_{1}=C_{c}R_{c}C_{c}^{H}+\sigma^{2}I.$$

Perform the EVD of $R-R_{1}$ as follows:

(47) $$R-R_{1}=U_{c,x}\Lambda_{c,x}U_{c,x}^{H}+U_{c,z}\Lambda_{c,z}U_{c,z}^{H}$$

where $U_{c,x}$ and $U_{c,z}$ are called the signal and noise subspaces associated with the signal eigenvalue matrix $\Lambda_{c,x}$ and noise eigenvalue matrix $\Lambda_{c,z}$, respectively. Similarly, we get the following relationship:

(48) $$C_{c}^{H}\left(\theta_{c,k},\beta_{c,k}\right)\;U_{c,z}U_{c,z}^{H}C_{c}\left(\theta_{c,k},\beta_{c,k}\right)\;=0,k=1,\ldots ,K_{c}.$$

and estimate the 2D directions-of-arrival (namely $\theta_{c,k}$ and $\beta_{c,k}$) of circular signals in the same way as the 2D directions-of-arrival of noncircular signals.

At this point, the 2D directions-of-arrival of noncircular and circular signals have been achieved by the TSRARE algorithm. The simple summary of the TSRARE algorithm is as follows.

**Step** **1:** Calculate R and $R^{\prime }$ with the observed data Z(t) with Equations (31) and (32);
**Step** **2:** Perform the SVD of $R^{\prime }$ to get $U_{n,2}$ using Equation (33);
**Step** **3:** Calculate the roots of $\det[T\left(b_{n,k}\right)]$ associated with noncircular signals from Equation (34) to Equation (39);
**Step** **4:** Attain $\theta_{n,k}$ and $\beta_{n,k}$ using Equations (40) and (42);
**Step** **5:** Construct $R_{1}$ with the estimate $R_{n,1}$ and $C_{n}$;
**Step** **6:** Perform the EVD of $R-R_{1}$ to get $U_{c,z}$ using Equation (47);
**Step** **7:** Repeat **Step 3** to **Step 4** for the $\theta_{c,k}$ and $\beta_{c,k}$.

**Remark 1**: In practice, it can be noted that only a finite number of observed data is available. Thus, $R_{xx}$, $R_{yx}$, $R_{xx}^{\prime }$, $R_{yx}^{\prime }$, R, and $R^{\prime }$ must be estimated by ${\hat{R}}_{\mathrm{xx}}$ = $\frac{1}{L}\sum_{l=1}^{L}X\left(l\right)X^{H}\left(l\right)$, ${\hat{R}}_{\mathrm{yx}}$ = $\frac{1}{L}\sum_{l=1}^{L}Y\left(l\right)X^{H}\left(l\right)$, ${\hat{R}}_{\mathrm{xx}}^{\prime }$ = $\frac{1}{L}\sum_{l=1}^{L}X\left(l\right)X^{T}\left(l\right)$, ${\hat{R}}_{\mathrm{yx}}^{\prime }$ = $\frac{1}{L}\sum_{l=1}^{L}Y\left(l\right)X^{T}\left(l\right)$, $\hat{R}$ = $\frac{1}{L}\sum_{l=1}^{L}Z\left(l\right)Z^{H}\left(l\right)$, and ${\hat{R}}^{\prime }$ = $\frac{1}{L}\sum_{l=1}^{L}Z\left(l\right)Z^{T}\left(l\right)$. When less observed data are available, such that the estimated covariance matrices are no longer strictly diagonal matrices, this leads to the estimation performance reduction in the small 2D angle separation condition.

**Remark 2**: As we know, one of the awkward problems about 2D direction-of-arrival estimation is the pair situation, which causes severe estimation error without exact pair process. However, the two proposed methods can pair the 2D directions-of-arrival of mixed signals automatically. This is because, in the two-stage estimation procedures, the TSDOAM method pairs the 2D directions-of-arrival by performing one EVD whose eigenvalues and eigenvectors are a one-to-one correspondence relationship, while the TSRARE method pairs them by decoupling the 2D directions-of-arrival into two successive 1D processes.

## 4. Location Discussion and Analysis

The maximum number of sources to estimate is analyzed in this section by the two proposed methods. Since the two proposed methods estimate the mixed signals separately, and the number of sources can be resolved is related to the dimensions of the elliptic auto-covariance matrix $R_{xx}^{\prime }$ (see Equation (8)) and the conjugated covariance matrix R (see Equation (31)), it follows that $M>max\left\{K_{n},K_{c}\right\}$ with the TSDOAM method and $2M>max\left\{K_{n},K_{c}\right\}$ with the TSRARE method must be satisfied, respectively, so as to resolve all noncircular and circular signals, while Yin’s [15] and Xia’s [16] method estimate them simultaneously, so $M>(K_{n}+K_{c})$ and $2M>(K_{n}+K_{c})$ should be satisfied, respectively. In other words, the maximum detectable signals of the TSDOAM method that are detected by two ULAs with 2M elements are (M-1) noncircular signals plus (M-1) circular signals, namely (2M-2), and the TSRARE method can detect (2M-2) noncircular signals plus (2M-2) circular signals, namely (4M-4); while Yin’s and Xia’s method can distinguish (M-1) and (2M-2) mixed signals, respectively. Therefore, the TSDOAM and TSRARE methods can detect twice the mixed signals as Yin’s and Xia’s method, respectively. In addition, the TSRARE method can identify twice the signals as the TSDOAM method.

## 5. Simulation Results

In this section, some simulation results are presented to show the performance of the TSDOAM and TSRARE methods, compared with the existing methods, which include Yin’s method and Xia’s method. Assume two ULAs with each array consisting of omnidirectional sensors spaced by a half wavelength of the mixed noncircular and circular signals; the distance between the two ULAs is spaced by a half wavelength as well. Additionally, the noncircular signals employ BPSK-modulated sources, while the circular signals are QPSK-modulated sources for the simulation.

### 5.1. 2D Direction-of-Arrival Estimation Performance

**Figure 2:**
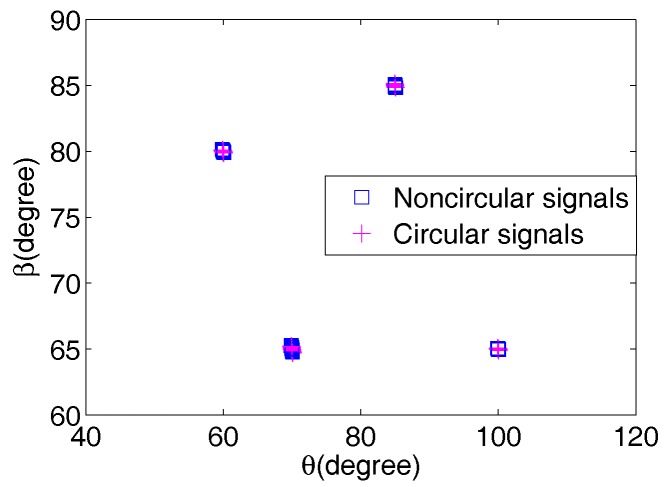
The 2D direction-of-arrival estimation scattergram of the two-stage rank reduction (TSRARE) method.

In this part, the maximum detectable 2D directions-of-arrival are investigated. Four BPSK signals with $\theta_{n,k}$ and $\beta_{n,k}$ impinge from $\left\{70^{\circ },60^{\circ },85^{\circ },100^{\circ }\right\}$ and $\left\{65^{\circ },80^{\circ },85^{\circ },65^{\circ }\right\}$, separately, and four QPSK signals are emitted from the same 2D directions-of-arrival. The number *M* of isotropic sensors of each array is three. Figure 2 plots the paired results of eight radiating signals from 50 Monte Carlo trials with the signal-to-noise ratio (SNR) set at 30 dB and snapshots *L* = 2000, which show that the 2D directions-of-arrival of eight (4M-4 = 8) signals are paired correctly with the TSRARE method; even a common β is shared in both noncircular and circular signals. However, the TSDOAM method and the methods in [15,16] can detect up to four (2M-2 = 4) signals, four (2M-2 = 4) signals, and two (M-1 = 2) signals, respectively, which fail to distinguish the mixed eight signals due to the limited array elements.

### 5.2. The Effect of SNR

In this subsection, we compare the 2D direction-of-arrival performance of the TSDOAM and TSRARE methods versus SNR with existing methods in [15,16]. Furthermore, the average root mean square error (ARMSE) is defined for precision evaluation as:

(49) $$\mathrm{ARMSE}=\sqrt{\sum_{k\;=\;1}^{K}\sum_{q=1}^{Mc}[({\hat{\zeta }}_{qk}\;-\;\zeta_{k}{)}^{2}]}$$

where $\zeta_{k}$ stands for $\theta_{k}$ or $\beta_{k}$, and ${\hat{\zeta }}_{qk}$ is the parameter to be estimated for ${\hat{\theta }}_{k}$ or ${\hat{\beta }}_{k}$, while $M_{c}$ denotes the number of Monte Carlo runs.

**Figure 3:**
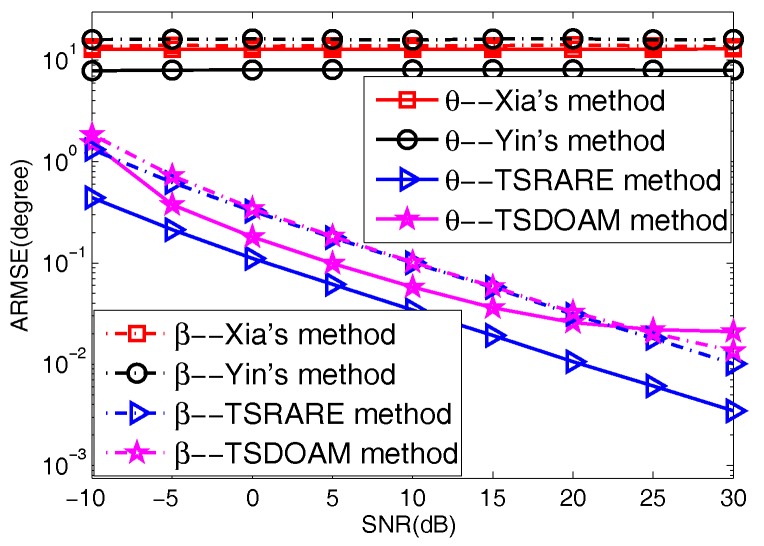
The average root mean square error (ARMSE) versus signal-to-noise ratio (SNR). TSDOAM: two-stage direction-of-arrival matrix.

Two BPSK signals together with two QPSK signals incoming from $\left(75^{\circ },50^{\circ }\right)$, $\left(100^{\circ },65^{\circ }\right)$ and $\left(75^{\circ },50^{\circ }\right)$, $\left(100^{\circ },65^{\circ }\right)$, respectively, impinge onto the ULAs with each array having five omnidirectional sensors. The snapshots of this test are set to 500, and the variable SNR of the four incident signals varies from −10 dB to 30 dB. The ARMSE of the four methods derived from the 2000 trials are given in Figure 3. It can be seen from Figure 3 that Yin’s method and Xia’s method fail to work; however, the two proposed methods perform well with increasing SNR. This is because the two proposed methods estimate the 2D directions-of-arrival of noncircular and circular signals separately based on the circularity difference between noncircular and circular signals rather than the direction-of-arrival difference utilized in Yin’s and Xia’s methods. Moreover, the TSRARE method has a lower ARMSE than the TSDOAM method, which results from the fact that the array aperture is fully utilized with the TSRARE method during the two-stage estimation for the BPSK and QPSK signals.

### 5.3. The Effect of Snapshots

**Figure 4:**
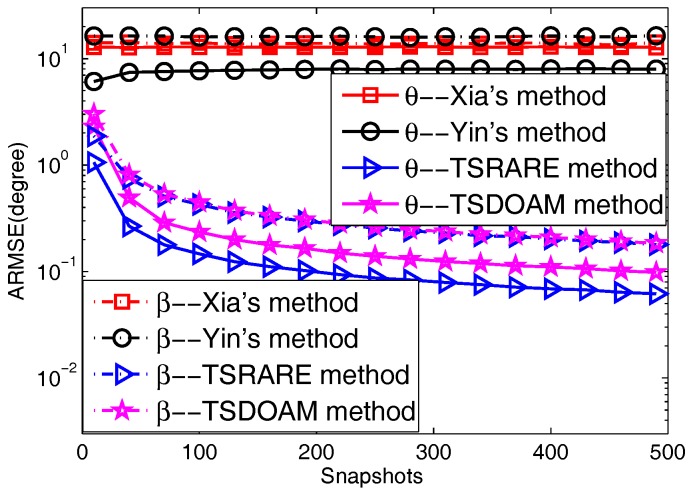
The ARMSE versus Snapshots.

The 2D direction-of-arrival performance of the TSDOAM and TSRARE methods versus snapshots with Yin’s and Xia’s methods is verified in this part. The simulation parameters are the same as Experiment 2, except the SNR is fixed at 5 dB, and the number of the collected snapshots varies from 10 to 490. At each snapshot setting, 2000 independent runs are executed for each method to obtain their 2D direction-of-arrival estimation in the statistical sense, whose results are given in Figure 4. Similar conclusions and reasons can be drawn from Figure 4 that as snapshots are increasing, the curves of the two proposed methods work well, while those of Yin’s and Xia’s methods remain unchanged. In addition, the TSRARE method has better estimation performance than the TSDOAM method in both 2D directions-of-arrival.

### 5.4. The Effect of Angle Separation

In this part, we testify to the 2D direction-of-arrival estimation performance of the two proposed methods coupled with Yin’s and Xia’s methods versus angular separation. The number of each array of the two ULAs is also five. Additionally, four signals consist of two BPSK signals and two QPSK signals with $\theta_{k}$ and $\beta_{k}$ incoming from $\left\{70^{\circ },100^{\circ },{\left(100+\Delta \right)}^{\circ },{\left(70+\Delta \right)}^{\circ }\right\}$ and $\left\{50^{\circ },85^{\circ },{\left(85+\Delta \right)}^{\circ },{\left(50+\Delta \right)}^{\circ }\right\}$, separately, and Δ is varied from 0 to 12. In addition, the SNR is fixed at 15 dB, and the snapshots *L* = 300.

**Figure 5:**
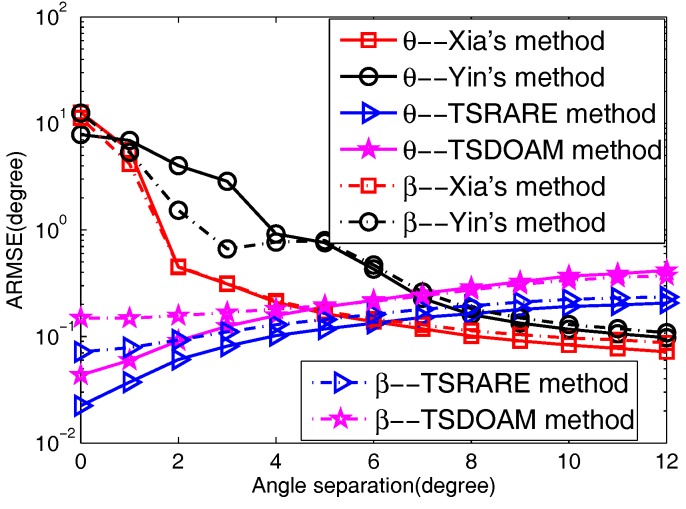
The ARMSE versus angular separation.

The ARMSE versus angular separation is shown in Figure 5 with 2000 Monte Carlo trials used. From Figure 5, it can be seen that the two proposed methods outperform Yin’s and Xia’s methods in both 2D small angle separations; however, with the angle separation increasing, the 2D direction-of-arrival estimation performance achieved by the two proposed methods is inferior to that by Yin’s and Xia’s methods. This is because in small angle separation,

the 2D direction-of-arrival information of circular signals included in the elliptic covariance matrix can be equivalent to that of noncircular signals, due to the fact that the 2D direction-of-arrival of the circular signal is close to that of the noncircular signal; thus, the estimation precision of the noncircular signals can be improved. A similar reason is suitable for the improved 2D direction-of-arrival estimation accuracy of circular signals; while Yin’s and Xia’s methods estimate the 2D direction-of-arrival of noncircular and circular signals simultaneously, which are based on the direction-of-arrival difference of noncircular and circular signals that inevitably gives rise to the degradation of the performance in small 2D direction-of-arrival separation [35]. As for large 2D direction-of-arrival separation, the estimation performance of the two proposed methods behaved with lower accuracy than Yin’s and Xia’s methods. The reason is that in the condition of a few snapshots, the incident mixed signals’ covariance matrix and elliptic covariance matrix are not strictly diagonal matrices, which results in making it difficult to separate the mixed signals from the two-stage estimation procedures. Furthermore, the TSRARE method performs better than the TSDOAM method all the way, with the full usage of array elements in the two-stage estimation procedures.

## 6. Conclusions

Two novel 2D direction-of-arrival estimation methods, named, respectively, as the TSDOAM and TSRARE methods for mixed noncircular and circular signals’ estimation with two parallel ULAs are presented in this paper. The direction-of-arrival circularity difference rather than the direction-of-arrival difference between the noncircular and circular signals for the 2D directions-of-arrival’ estimation is utilized in the two proposed methods. The explicit derivation of the two proposed methods is described, and the maximum number of incident signals of the two proposed methods is analyzed, which shows that the detected number of signals is more than that of the array elements compared to the conventional methods. Simulation results demonstrate the usefulness of the two proposed methods.

## Abbreviations

The following abbreviations are used in this manuscript:

DOA	direction-of-arrival
DOAs	directions-of-arrival
TSDOAM	two-stage direction-of-arrival matrix
TSRARE	two-stage rank reduction
ERARE	extended rank reduction
ULAs	uniform linear arrays
ESPAR	electronically steerable parasitic antenna radiator
EVD	eigen-value decomposition
SVD	singular-value decomposition
MUSIC	multiple signal classification
SNR	signal-to-noise ratio
ARMSE	average root mean square error
CRB	Cramér–Rao bound
